# Brick-and-mortar food retail implementation of a nutrition incentive program using Electronic Benefit Transfer integration in Southern California

**DOI:** 10.1093/tbm/ibag020

**Published:** 2026-05-11

**Authors:** Bailey Houghtaling, James P Marriott, Amy L Yaroch, Joe Prickitt, Blanca Melendrez, Karemi Alvarez, Nancy Knauer, Carmen Byker Shanks

**Affiliations:** Center for Nutrition & Health Impact, Omaha, NE, United States; Department of Human Nutrition, Foods, and Exercise, Virginia Tech, Blacksburg, VA, United States; Center for Nutrition & Health Impact, Omaha, NE, United States; Center for Nutrition & Health Impact, Omaha, NE, United States; Center for Community Health, Altman Clinical & Translational Research Institute, University of California San Diego, San Diego, CA, United States; Center for Community Health, Altman Clinical & Translational Research Institute, University of California San Diego, San Diego, CA, United States; Center for Community Health, Altman Clinical & Translational Research Institute, University of California San Diego, San Diego, CA, United States; Mother’s Nutritional Center, Santa Fe Springs, CA, United States; Center for Nutrition & Health Impact, Omaha, NE, United States

**Keywords:** food security, access to healthy foods, supplemental nutrition assistance program, implementation science

## Abstract

**Background:**

Nutrition incentive programs (NIPs) that integrate fruit and vegetable (FV) incentives into Supplemental Nutrition Assistance Program (SNAP) Electronic Benefit Transfer (EBT) systems have strong potential for scaling in the United States, yet little is known about implementation from brick-and-mortar food retailers’ perspectives.

**Purpose:**

To examine brick-and-mortar food retailers’ perceptions and experiences with an NIP using SNAP EBT integration for FV incentives at the point of sale.

**Methods:**

Guided by the Inner Context of the Exploration, Preparation, Implementation, Sustainment (EPIS) framework, an implementation science evaluation was conducted among 79 independent chain grocers implementing the NIP in Southern California. Leadership and staff were invited to complete an online survey (89% response rate) using validated tools (e.g. Implementation Leadership Scale), expert-developed items, and open-ended questions. The analysis included descriptive statistics and inferential tests that assessed response differences between leadership and staff.

**Results:**

Of 538 respondents, most were female (76%) and Latino/a (94%). Perceptions and experiences with the NIP were favorable across EPIS Inner Context constructs, with some differences noted. Leadership support for implementation was rated highly overall, though staff scored items on supportive and knowledgeable leadership lower compared to leadership. While highly satisfied with NIP implementation overall, open-ended responses highlighted opportunities to optimize implementation across stores.

**Conclusions:**

Nutrition incentive programs integrating FV incentives into SNAP EBT systems appear well-suited to the brick-and-mortar food retail context. Findings can inform the design and testing of implementation strategies to support sustainment and scaling for population-level impact locally and nationally.

## Teaser

This study explored how brick and mortar food retailers experienced the rollout of one of the first EBT-integrated NIPs in the United States, with results identifying alignment as well as opportunities to support broader implementation goals.

Implications

- **Practice:** Collaborate directly with brick-and-mortar food retailers to build trust and capacity for successful implementation of Electronic Benefit Transfer (EBT)-integrated nutrition incentive programs (NIPs).
- **Policy:** Allocate funding and technical support to address retailer-identified barriers and streamline the scaling of EBT-integrated NIPs.
- **Research:** Develop and test targeted implementation strategies to support brick-and-mortar food retailers to adopt, implement, and sustain NIPs integrated within EBT systems.

## Background

Several factors contribute to Americans’ lower-than-recommended intake of fruits and vegetables (FVs) [1], including limited access to fresh produce, affordability, and competing priorities for household budgets [2, 3]. Challenges are especially pronounced among customers with low income [4]. The Supplemental Nutrition Assistance Program (SNAP), the largest federal assistance program in the United States for individuals and families facing financial hardship, is strongly linked to improved poverty rates, food security, and positive health outcomes [5]. However, even with SNAP, Americans with low income struggle to afford nutritious foods like FVs [5, 6].

Nutrition incentive programs (NIPs) are a promising public health strategy to enhance SNAP populations’ purchasing power for FVs [7, 8]. A typical NIP offers matched financial incentives for FVs (e.g. spend $10, receive $10) to participating SNAP populations at the point of purchase. Although how NIPs are designed and implemented throughout the United States varies, including by factors such as incentive amount, eligible purchases that activate incentives (e.g. FVs versus any SNAP-eligible food item), eligible produce for incentive redemption (e.g. fresh FVs only versus allowing frozen and canned FVs), the incentive delivery and redemption mechanism (e.g. loyalty cards, automatic discounts, vouchers, tokens, coupons), as well as the participating retail venues (e.g. farmers market, brick-and-mortar food retail) where incentives can be redeemed [9, 10].

Recently practitioners have begun exploring NIP models that use SNAP Electronic Benefit Transfer (EBT) systems as the incentive delivery/redemption mechanism given a few potential benefits [11–16]. Use of EBT systems in NIPs has the potential to promote scalability given the existence of the technology in all US SNAP-authorized venues; reduce implementation burden through automatic incentive earning and redemption, especially compared to more manual procedures like vouchers or coupons; and improve NIP program reach through the automatic enrollment of eligible SNAP populations. However, there is limited to no implementation evidence available from key partners’ perspective, including brick-and-mortar food retailers.

Implementation science, the study of how to translate evidence into every day practice [17], provides a useful frame to advance knowledge and action in this regard by focusing on factors such as leadership support, alignment with business priorities, implementation fidelity, adequate staffing, and brick-and-mortar food retailer buy-in to promote NIP implementation, sustainment, and scaling [18–20]. Prior literature reviews highlight the complexity of public health interventions in brick-and-mortar food retail settings more broadly [21, 22]. Specific to traditional NIPs, there is limited insight into brick-and-mortar food retailers’ implementation successes/challenges, especially using robust implementation science frameworks [23–25]. Further, while some evidence exists for farmers market settings [16], there is no evidence on NIPs that leverage EBT systems in the brick-and-mortar food retail context, given their novelty. This implementation science evaluation aimed to fill critical gaps to inform the implementation, sustainment, and scaling of NIPs that leverage EBT systems in a complex and critical setting for consumer health promotion.

## Purpose

The aim of this study was to examine brick-and-mortar food retailers’ perceptions and experiences with an NIP using SNAP EBT integration for FV incentives at the point of sale. By doing so, the study team was interested in informing future directions for implementation research and practice to support wide scale implementation and impact of NIPs that leverage EBT systems.

## Methods

A cross-sectional, quantitative implementation science evaluation was carried out following the Exploration, Preparation, Implementation, Sustainment (EPIS) framework [18, 19]. Results about the implementation of the CalFresh Fruit & Vegetable EBT Pilot Project (Pilot Project) from the perspective of brick-and-mortar food retailers are presented herein. Findings pertaining to Pilot Project perspectives and experiences among farmers market managers/vendors and program participants are presented elsewhere [16]. The STROBE (STrengthening the Reporting of OBservational studies in Epidemiology) guidance informed manuscript reporting [26].

The Pilot Project was implemented through a partnership between the California Department of Social Services, the University of California San Diego (UCSD) Center for Community Health, and Mother’s Nutritional Center (MNC). In collaboration with UCSD and MNC, a team from the Center for Nutrition & Health Impact (CNHI) led the external evaluation in the brick-and-mortar food retail context. CNHI is the lead organization for the Gus Schumacher Nutrition Incentive Program (GusNIP) Nutrition Incentive Program Training, Technical Assistance, Evaluation, and Information Center (NTAE) that supports NIP grantees funded by the US Department of Agriculture National Institute of Food and Agriculture. GusNIP is the largest funder of NIPs in the United States [27], although EBT integration is not currently supported under this mechanism.

Overarching Pilot Project implementation and evaluation plans were reviewed by the UCSD Institutional Review Board (IRB), and information about the evaluation plan inherent to this study was submitted to the University of Nebraska Medical Center IRB. In both instances, this research was determined to “not constitute human subject research” in accordance with US Department of Health and Human Services regulations for the protection of human subjects in research (45 CFR 46) and, therefore, did not require IRB review. Nonetheless, the study team sought informed consent for survey participation.

### Conceptual framework

EPIS is a process and determinant framework that was created in 2011 [18] and updated in 2019 [19]. Its use guides evidence-based intervention phases (Exploration, Preparation, Implementation, and Sustainment) and the factors that influence adoption, implementation, and/or sustainment across these phases [18, 19]. EPIS has been previously defined within the context of healthy food retail strategies, such as NIPs [20]. To limit survey burden, the EPIS Inner Context constructs were primarily used to guide the study evaluation, given its focus on the organizational context where implementation occurs (i.e. MNC stores), including Leadership, Organizational Characteristics, Quality and Fidelity Monitoring/Support, Organizational Staffing Processes, and Individual Characteristics. See Houghtaling *et al.* 2023 [20] for definitions of these constructs specific to the brick-and-mortar food retail setting. Other EPIS constructs include Bridging Factors (see below information about the NIP and implementation strategies used to support adoption and implementation), Innovation Factors (see information below about the NIP as well as Prickitt *et al.* 2025 [15]), and Outer Context or the factors external to the organization where implementation occurs. The study evaluation included a question about the zip code for implementing stores to understand a bit more about the local community context for the NIP.

### Nutrition incentive program

The Pilot Project helped in modernizing nutrition assistance by replacing vouchers, coupons, and loyalty cards with streamlined incentive integration into existing SNAP EBT systems [15]. Through the Pilot Project, all SNAP (CalFresh in California) participants earned a penny-for-penny rebate for eligible, fresh FV purchases made with EBT cards at retail venues. Up to 60 dollars per month could be earned by SNAP shoppers with incentives able to be redeemed for any SNAP-eligible food at any SNAP-authorized location. See Prickitt *et al.* 2025 [15] for more details about the Pilot Project specific to brick-and-mortar food retail settings in California.

To support Pilot Project adoption and implementation, UCSD and MNC delivered several implementation strategies, defined in implementation science as the methods used to translate evidence-based interventions into practice [28]. In consultation with partners, the CNHI team gathered information about these activities and aligned the description of strategies used with the Implementation Strategies Applied in Communities (ISAC) compilation [29]. ISAC was created to support the translation of evidence-based interventions in community settings [29], versus clinical contexts (i.e. the Expert Recommendations for Implementing Change compilation [30]), given a need for more community-specific methods in implementation science [31]. Implementation strategies used to support Pilot Project implementation in MNC stores are described in Supplementary Table S1.

### Brick-and-mortar food retailer

This evaluation focused on Pilot Project implementation in MNC stores (*n* = 79 at the time of the study). MNC is a family-owned independent grocery chain operating across Los Angeles, Orange, Riverside, San Bernardino, San Diego, and Imperial counties in Southern California [32]. MNC has served local communities for over 30 years and supports customers by prioritizing nutrition assistance services, including the Special Supplemental Nutrition Program for Women, Infants, and Children, SNAP, Summer EBT, and the +ADD MILK program which offers eligible customers a dollar-for-dollar match for nonfat, 1%, and 1% lactose-free milk. The MNC model is characterized as “pharmacy-style” that combines self-service and behind-the-counter order fulfillment to enhance customer support [32].

### Survey measures

Survey questions aimed to assess MNC retailers’ perceptions and experiences with Pilot Project implementation following EPIS Inner Context constructs [18, 19]. The study team prioritized use of pragmatic measures in the peer-reviewed literature, when available. For the Leadership construct, the Implementation Leadership Scale (ILS) was used [33]. The ILS is a validated 12-item tool that assesses four aspects of leadership support for evidence-based intervention implementation, including proactive, supportive, knowledgeable, and perseverant leadership. There are two versions of the ILS, one that has leadership rate themselves, and one that prompts staff to rate leadership [33]. Given that the ILS was originally developed in a healthcare context, the study team, including evaluation, practice, and retail partners, adapted terminology to better fit the brick-and-mortar food retail setting. A separate publication is forthcoming that used confirmatory and multiple group factor analyses to evaluate the suitability of the adapted ILS to measure leadership support for NIP implementation in the brick-and-mortar food retail context; findings were favorable.

Additionally, two questions from a healthy food retail survey used in Aboriginal and Torres Strait Islander communities [34] were included. One question focused on store-community relationships to support healthy food retail programs (Organizational Characteristics construct), and another focused on staff capacity to support programming (Organizational Staffing Processes construct). A general measure of satisfaction was also included by using the Net Promoter Score (to reflect all EPIS Inner Context constructs), which is a one-item question that was used to assess MNC retailers’ likelihood of recommending the Pilot Project on a 0–10 rating scale (higher values indicating higher satisfaction) [35].

Remaining survey questions were selected from an existing internal resource of evaluation measures for GusNIP NIPs created by NTAE partners at the National Grocers Association Foundation or were developed by the study team. Existing literature about factors that influence NIP implementation in the brick-and-mortar food retail setting was reviewed to ensure the survey covered all relevant topics in accordance with EPIS Inner Context constructs [23, 24]. The survey also included social, demographic, and role-related questions as well as a question about the zip code of the implementing store to allow the study team to refer to secondary data about local community characteristics. Most survey items included select/multi-select or Likert scale response options, with all questions including “prefer not to answer” and/or “I don’t know/not applicable.” Several open-ended questions were also included to gather broader, optional feedback. Skip logic in Qualtrics ensured role-relevant questions were used among three survey populations: higher-level leadership (30 items), store-level leadership (39 items), and store staff (39 items). The full survey is available in Supplementary File S1.

### Sample and recruitment

All MNC retailers (*n* = 620 at the time of the study) across 79 implementing stores were eligible to participate. The three groups of MNC retailers were included: (i) higher-level leadership or the Owner, Directors, District Managers, and Marketing/Outreach Managers; (ii) store-level leadership or the Managers, Assistant Managers, 3rd Persons (i.e. an employee one step below assistant manager who mainly assists in managerial tasks), and Crew Leaders (i.e. an employee one step below 3rd persons who is a cashier lead and helps in cashiering and breaks/lunch coverage); and (iii) store staff or the Cashiers, Produce Attendants, Stockers, and Marketing/Outreach staff. To recruit for survey participation, an electronic store memo with a link to the online Qualtrics survey was distributed through established MNC communication channels on 26 March 2024. Two follow-up reminders were sent using the same method before the survey closed on 8 April 2024. The evaluation timeline was shortened due to an earlier-than-expected spend-down of project funds attributed to customer demand for the NIP. MNC retailers were encouraged to complete the survey during shift hours and using store computers. Fresh juice was offered to encourage optional survey participation. A total of 552 survey responses were received, yielding an 89.0% response rate.

### Data analysis

Survey responses were reviewed for illogical or incomplete data, resulting in the removal of 14 cases from the final analytic sample (*n* = 538). Survey responses were used to create scores, when applicable. The ILS was scored using established procedures and ranged from 0 to 4, with higher values for items indicating greater leadership support [33]. Other Likert-style items were scored similarly for consistency. The Net Promoter Score also followed standard scoring criteria, with respondents categorized as promoters (ratings of 9–10), passives (7–8), or detractors (≤6); the total score had a possible range of −100 to 100 and was calculated by subtracting the percentage of detractors from the percentage of promoters [35].

All descriptive and inferential statistics were calculated using R (version 4.4.1). Missing data and responses marked “I don’t know/not applicable” or “Prefer not to answer” were excluded from item-level analyses. A goal of inferential analyses was to understand if retail leadership and staff responses diverged. A small body of literature highlights the potential for differences in perception/experience between these populations, which may influence healthy food retail strategy buy-in, implementation, and sustainment [21, 36–38]. Chi-square and Mann–Whitney *U* tests for nonparametric data (α = 0.05) were used to compare survey responses between store leadership and staff. The *P*-values for Mann–Whitney *U* tests are shown in the results text and tables; however, means and standard deviations are reported in place of medians and interquartile ranges (IQRs) to improve the interpretation of the results as medians and IQRs often masked notable differences.

Further, store zip codes provided by respondents were matched to the Social Deprivation Index to characterize the local socioeconomic context [39]. Racial and ethnic composition data were also matched to zip codes using 2020 American Community Survey 5-year estimates [40]. Response data for the open-ended survey questions were analyzed using general qualitative data analysis principles [41, 42] to code and categorize data into themes with two-person agreement.

## Results

### Sample characteristics

Most MNC retailers who participated in the survey held store-level staff positions, with Cashiers being the largest group (*n* = 193, 36%) (Table 1). Crew Leaders were the most represented among the store-level leadership positions (*n* = 107, 20%). MNC retailers’ median age was 27.5 years, with store-level leadership slightly older than staff. The sample was predominantly female (76%) and of Hispanic, Latino/a, or Spanish origin (94%). Employment duration varied, with many employed for >1 month but <1 year (*n* = 149, 30%) or for 10 or more years (*n* = 123, 23%). Leadership and management tended to have longer tenure than staff (Table 1).

**Table 1:** Characteristics of MNC leadership and staff implementing a nutrition incentive program using EBT integration at the point of sale across 79 stores in California, 2024.

Retailer characteristics	Total *n* = 538 (%)	Store leadership *n* = 306 (%)	Store staff *n* = 232 (%)
**Position title**			
Cashier	193 (35.9)		
Crew Leader	107 (19.9)		
Manager	74 (13.8)		
Assistant Manager	69 (12.8)		
3rd Person	24 (4.5)		
Stocker	21 (3.9)		
District Manager	16 (3.0)		
Marketing/Outreach Manager	11 (2.0)		
Produce Attendant	11 (2.0)		
Marketer/Outreach	7 (1.3)		
Director	4 (0.7)		
Owner	1 (0.2)		
**Age**			
(Median [IQR])	27.5 [21–35]	31.0 [25–39]	22.0 [19–29]
**Sex**			
Female	410 (76.2)	247 (80.7)	163 (70.3)
Male	125 (23.2)	59 (19.3)	66 (28.4)
Did not report female or male	2 (0.4)	0 (0)	2 (0.9)
Prefer not to answer	1 (0.2)	0 (0)	1 (0.4)
**Race/Ethnicity**			
American Indian or Alaska Native, non-Hispanic	1 (0.2)	0 (0.0)	1 (0.4)
Asian, non-Hispanic	1 (0.2)	1 (0.4)	0 (0.0)
Black or African American, non-Hispanic	12 (2.2)	5 (1.6)	7 (3.0)
Hispanic, Latino/a, or Spanish origin	508 (94.4)	289 (94.4)	219 (94.4)
White, non-Hispanic	7 (1.3)	5 (1.6)	2 (0.9)
Some other race	2 (0.4)	0 (0.0)	2 (0.9)
Prefer not to answer	7 (1.3)	6 (2.0)	1 (0.4)
**Length of employment**			
<1 month	18 (3.3)	0 (0)	18 (7.8)
>1 month but <1 year	149 (27.7)	9 (2.9)	140 (60.3)
1–2 years	86 (16.0)	45 (14.7)	41 (17.7)
2–3 years	42 (7.8)	30 (9.8)	12 (5.2)
3–5 years	36 (6.7)	27 (8.8)	9 (3.9)
5–10 years	79 (14.7)	75 (24.5)	4 (1.7)
10+ years	123 (22.9)	118 (38.6)	5 (2.2)
Prefer not to answer	5 (0.9)	2 (0.7)	3 (1.3)

Regarding the local context of NIP implementing stores, the Social Deprivation Index ranged from 29 to 100 (average 84), indicating high area disadvantage (data not shown). Most implementing stores were located in communities where 67% of the population identified as Hispanic or Latino/a, 14% as non-Hispanic White, 9% as non-Hispanic Black or African American, and 8% as non-Hispanic Asian (data not shown).

### Implementation satisfaction

The Net Promoter Score attributed to the Pilot Project was 89 out of 100 (data not shown), indicating high satisfaction.

### Leadership

Leadership support for Pilot Project implementation was rated highly among both store leadership and staff, with some differences between groups (Table 2). Store leadership rated themselves higher compared to staff ratings for store leadership on the “Supportive Leadership” scale overall (3.65 ± 0.58 vs. 3.48 ± 0.55; *P *< .001), and on each item: recognizing/appreciating employee efforts (3.70 ± 0.60 vs. 3.47 ± 0.66; *P *< .001), supporting learning about the program (3.64 ± 0.63 vs. 3.48 ± 0.60; *P *< .001), and supporting program implementation (3.63 ± 0.62 vs. 3.48 ± 0.57; *P *< .001). Store leadership also rated themselves higher on one item from the “Knowledgeable Leadership” scale, regarding knowledge about the program (3.64 ± 0.64 vs. 3.53 ± 0.67; *P* = .016) (Table 2).

**Table 2:** Implementation leadership support for the implementation of a nutrition incentive program using EBT integration at the point of sale across 79 MNC stores in Southern California, 2024.

ILS^a^	Store leadership	Store staff	*P*-value
	Mean ± SD (total min, total max)	Mean ± SD (total min, total max)	
**Proactive leadership^b^**	3.41 ± 0.67 (0, 12)	3.41 ± 0.57 (6, 12)	.47
Provision of tools and resources	3.53 ± 0.77 (0, 4)	3.57 ± 0.68 (0, 4)	.91
Removal of implementation obstacles	3.18 ± 0.97 (0, 4)	3.10 ± 1.00 (0, 4)	.29
Establishing clear standards	3.52 ± 0.65 (0, 4)	3.49 ± 0.65 (0, 4)	.59
**Knowledgeable leadership^c^**	3.65 ± 0.56 (0, 12)	3.57 ± 0.55 (3, 12)	.054
Knowledgeable about the pilot project	3.64 ± 0.64 (0, 4)	3.53 ± 0.67 (0, 4)	**.016** ^g^
Ability to answer questions	3.64 ± 0.59 (0, 4)	3.57 ± 0.57 (0, 4)	.071
Knowing what they are talking about	3.66 ± 0.58 (0, 4)	3.59 ± 0.56 (0, 4)	.078
**Supportive leadership^d^**	3.65 ± 0.58 (0, 12)	3.48 ± 0.55 (5, 12)	**<.001** ^g^
Recognizing and appreciating employee efforts	3.70 ± 0.60 (0, 4)	3.47 ± 0.66 (0, 4)	**<.001** ^g^
Supporting employee efforts to learn more about the pilot project	3.64 ± 0.63 (0, 4)	3.48 ± 0.60 (1, 4)	**<.001** ^g^
Supporting employee efforts to carry out the pilot project	3.63 ± 0.62 (0, 4)	3.48 ± 0.57 (1, 4)	**<.001** ^g^
**Perseverant leadership^e^**	3.51 ± 0.58 (0, 12)	3.48 ± 0.52 (4, 12)	.33
Persisting through the ups and downs	3.49 ± 0.64 (0, 4)	3.46 ± 0.57 (1, 4)	.26
Continuing through the challenges	3.50 ± 0.65 (0, 4)	3.48 ± 0.60 (0, 4)	.44
Responding to critical issues	3.49 ± 0.62 (0, 4)	3.49 ± 0.54 (2, 4)	.61
**Total ILS score^f^**	3.57 ± 0.53 (0, 48)	3.50 ± 0.47 (22, 48)	**.048** ^g^

a Response options for each of the ILS items ranged from 0 (completely disagree) to 4 (completely agree) [33]; therefore, higher mean values indicated more positive leadership support.

b Overall subscale mean calculated as an average of the three subscale items. Those with missing data for any item within the proactive leadership dimension were excluded from the analysis. MNC leadership *n* = 303; MNC staff *n* = 208.

c Overall subscale mean calculated as an average of the three subscale items. Those with missing data for any item within the knowledgeable leadership dimension were excluded from the analysis. MNC leadership *n* = 304; MNC staff *n* = 231.

d Overall subscale mean calculated as an average of the three subscale items. Those with missing data for any item within the supportive leadership dimension were excluded from the analysis. MNC leadership *n* = 304; MNC staff *n* = 230.

e Overall subscale mean calculated as an average of the three subscale items. Those with missing data for any item within the perseverant leadership dimension were excluded from the analysis. MNC leadership *n* = 291; MNC staff *n* = 220.

f Overall ILS scale mean calculated as an average of the four subscale means. Those with missing data for any item were excluded. MNC leadership *n* = 289; MNC staff *n* = 202.

g Bolded values indicate statistical significance.

### Organizational characteristics

All higher-level leadership (*n* = 5) agreed that the cost and time required by the organization to implement the Pilot Project were worthwhile (3.60 ± 0.55; range = 3–4). Their primary motivations for participation included benefiting customers (100%) and engaging in a new retail initiative (80%). Additional reasons included increasing sales of FVs (60%) and boosting overall sales (40%). Further, MNC’s relationship with local communities to facilitate Pilot Project implementation was rated favorably overall (3.67 ± 0.56, range = 0–4, *n* = 537), with a difference found between store leadership and staff (3.70 ± 0.58, range = 0–4, *n* = 305 vs. 3.63 ± 0.53, range = 2–4, *n* = 232; *P* = .022). Most MNC retailers did not think changes were needed to better align the Pilot Project with MNC’s mission or values (*n* = 419, 85.5%) or to better meet customer needs (*n* = 420, 84.5%); although, a greater proportion of those who noted changes were needed regarding mission/values (18.4% vs. 8.9%; *P* = .005) or customer needs (18.6% vs. 11.2%; *P* = .034) were store leadership compared to staff. Most MNC retailers did not think changes were needed to the Pilot Project to better align with store processes/procedures (*n* = 420, 84.5%), with no differences between groups.

### Quality and fidelity monitoring/support

Most MNC retailers reported a positive experience with marketing and promoting the Pilot Project (3.54 ± 0.68, range = 0–4, *n* = 524), with store-level leadership responding more favorably than staff (3.59 ± 0.70, range = 0–4, *n* = 296 vs. 3.48 ± 0.65, range = 2–4, *n* = 228; *P* = .010). The trainings, resources, and materials provided to MNC retailers to support Pilot Project implementation were regarded favorably overall (3.47 ± 0.67, range = 0–4, *n* = 528), with no differences between groups. Most MNC retailers reported not needing additional support for Pilot Project implementation (*n* = 445, 90.4%), also with no differences between groups identified.

### Organizational staffing processes

Overall, MNC retailers rated having enough staff with the right skills to support Pilot Project implementation highly (3.22 ± 0.86, range = 0–4, *n* = 529), with no differences identified between groups. Further, MNC retailers rated the ease of explaining how the Pilot Project works (3.05 ± 0.89, range = 0–4, *n* = 530), the types of FVs eligible for the Pilot Project (3.46 ± 0.69, range = 1–4, *n* = 529), and the differences between Pilot Project and other state/federal store programs (3.26 ± 0.79, range = 0–4, *n* = 523) highly overall, with no differences found between groups. The ease of handling Pilot Project transactions was also rated highly by MNC retailers in general (3.43 ± 0.69, range = 1–4, *n* = 514), with store-level leadership having higher ratings compared to staff (3.48 ± 0.67, range = 1–4, *n* = 299 vs. 3.34 ± 0.71, range = 1–4, *n* = 215; *P* = .018). MNC retailers reported fielding various customer questions about the Pilot Project, including the program logistics (87%), the program end (84%), and incentive/EBT balances (78%). Most MNC retailers noted that processing new Pilot Project customers required between 2 and 5 min (36.5%, *n* = 194) or between 30 s and 2 min (29.7%, *n* = 158) of extra time. A smaller proportion noted that >5 min (13.2%, *n* = 70) or <30 s were needed (8.3%, *n* = 44); some also reported that no extra time was required (6.6%, *n* = 35).

### Individual characteristics

Mother’s Nutritional Center retailers rated their experience with implementing the Pilot Project highly overall (3.47 ± 0.74, range = 0–4, *n* = 528), with higher ratings found among store-level leadership compared to staff (3.58 ± 0.70, range = 1–4, *n* = 297 vs. 3.34 ± 0.76, range = 0–4, *n* = 231; *P *< .001). In general, MNC retailers believed that it was important to support programs that promote FV access like the Pilot Project (3.66 ± 0.58, range = 0–4, *n* = 530), also with higher ratings among store-level leadership compared to staff (3.71 ± 0.56, range = 0–4, *n* = 301 vs. 3.60 ± 0.61, range = 0–4, *n* = 229; *P* = .008). MNC retailers also believed that participation in the Pilot Project provided an opportunity to give back to the community (3.54 ± 0.65, range = 0–4, *n* = 526), also with differences found between store-level leadership and staff (3.61 ± 0.60, range = 0–4, *n* = 299 vs. 3.45 ± 0.69, range = 0–4, *n* = 227; *P* = .004). Support for MNC’s participation in a program like the Pilot Project in the future was also rated highly in general (3.63 ± 0.63, range = 0–4, *n* = 528), with higher ratings also found among store-level leadership versus staff (3.69 ± 0.62, range = 0–4, *n* = 301 vs. 3.54 ± 0.64, range = 0–4, *n* = 227; *P *< .001).

### Open-ended survey responses

A total of 247 MNC retailers provided open-ended feedback in response to broad questions. Responses were grouped into eight themes that provided insight into MNC retailers’ perceptions and experiences with the Pilot Project. Full thematic details and supporting quotations are available in Supplementary Table S2. Most frequently, MNC retailers emphasized the Pilot Project’s role in supporting food and nutrition security in local communities and expressed a desire for its continuation. Additional themes offered nuanced feedback from MNC retailers’ that, if addressed, could optimize implementation across all sites, including: simplifying incentive/EBT balance tracking for customers; increasing customer awareness and understanding of the Pilot Project; revising incentive transaction rules for efficiency; improving staffing and store operations to support increased demand; addressing retailer-customer language barriers; expanding eligible purchases beyond fresh FVs to benefit all customers; and enhancing staff training and technical assistance strategies for the Pilot Project (Supplementary Table S2).

## Discussion

A cross-sectional, quantitative implementation science evaluation was carried out to understand brick-and-mortar retailers’ perceptions and experiences with implementing one of the first NIPs in the United States using EBT integration at the point of sale. Results can help frame opportunities to promote the adoption, implementation, sustainment, and scaling of novel NIPs in a complex health promotion setting. Results from this study suggest that NIPs using EBT integration for incentives are well suited for the brick-and-mortar retail context, given favorable views on leadership support, organizational fit, suitable supports to ensure delivery, sufficient staffing, and positive views among individual retailers. However, there were several results that could inform future implementation and scaling preparation to ensure optimization.

First, while the retailers in this study responded favorably to survey items across EPIS Inner Context constructs [18, 19], there was a trend in which store leadership responded slightly more favorably than staff for many items, suggesting somewhat less favorable perceptions/experiences among store-level staff who were the day-to-day implementors of NIPs with higher customer facing responsibilities. This trend was specifically reversed for perspectives on changes needed to better align the NIP with the stores’ mission/values and customers’ needs, where slightly more leadership believed that programmatic changes were needed compared to staff. While a small body of literature in the healthy food retail space has highlighted the existence of diverging views between various levels of retail leadership and staff [21, 36–38], there is no evidence on how such gaps translate to adoption, implementation, or sustainment barriers in this setting, which could limit scaling potential. Follow-up qualitative inquiry with leadership and staff implementors of the NIP could help to understand their views on and reasons for diverging perspectives, which could inform the development of robust implementation strategies that target leadership support. To further build the evidence base, attention to differences in retail leadership and staff perspectives and experiences with implementation is recommended for similar NIP initiatives using EBT integration that are currently occurring in several US locations [12–14].

Additional implementation insights from some food retailers were gathered in this study using broad open-ended survey questions. In alignment with the quantitative survey results, retailers who responded to these questions appreciated the NIP and its support of community food and nutrition security and wanted it to continue. At the same time, retailers provided insight into implementation challenges that, if addressed, may optimize NIP delivery across stores and promote scaling. Reported challenges were focused on NIP transaction process constraints; customer awareness and understanding of the NIP; communication challenges; staff and store capacity constraints, and limitations of the eligible (i.e. fresh) produce for the NIP. Overall, these positive and constructive sentiments largely aligned with qualitative findings among farmers market vendors/staff and customers regarding Pilot Project implementation in these settings, suggesting some experiential alignment across retail contexts [16]. This could indicate appropriateness for common implementation strategies [31] to be designed and tested to support the implementation of NIPs using EBT integration across farm and brick-and-mortar retail settings. However, more in-depth qualitative inquiry among brick-and-mortar food retail implementers of NIPs using EBT integration is needed to understand potential challenges more comprehensively.

Based on the results of this study, future directions for implementation research and practice include designing and testing preliminary implementation strategies detailed in the ISAC compilation to support the adoption, implementation, sustainment, and scaling of NIPs using EBT integration across retail contexts for SNAP customer benefit [29]. The ISAC strategies indicated for potential design and tailoring among practice, retail, and implementation science partners included: leverage funding sources to support continued NIP delivery; tailor recruitment strategies and materials to engage with customers in preferred languages; change adaptable NIP components (e.g. eligible produce) to meet customers’ and retailers’ needs; enhance store layouts or add equipment to support increased customer demand and transaction times; enhance staffing, which may include addressing leadership support and differences between leadership and staff; provide technical assistance; and encourage use of existing or new technology to support knowledge of NIP balances [29]. Decisions about whether strategies to support NIPs using EBT integration are applied program wide or for specific retail settings should be determined using collaborative implementation strategy design and specification methods at local levels [28, 43].

### Practice implications

The success of the Pilot Project in California has been leveraged by UCSD and MNC to build momentum for the sustainment and expansion of NIPs statewide. Pilot Project insights have also helped to advance US Department of Agriculture priorities by serving as a model for the development and implementation of innovative NIPs using EBT integration to improve reach and reduce burden. By aligning with federal priorities and demonstrating scaling potential, UCSD and MNC have positioned the Pilot Project as a powerful catalyst for expanding access to healthy food in areas of the country that could benefit from such supports through sustainable, technology-driven solutions. The results of this work are intended to guide similar work in other areas of the country and in partnership with an array of food retail business models.

### Strengths and limitations

This study leveraged implementation science and evaluation expertise and practice and retail partnerships to evaluate a novel NIP using EBT integration and, as such, provides a framework for building on this evidence base across contexts. Intentional use of an implementation science framework to guide the evaluation was another strength. Additionally, partners’ engagement throughout the evaluation process was attributed to the excellent response rate (89%) rarely captured in survey research. However, there are several limitations to consider.

Most of the survey sample was of Hispanic, Latino/a, or Spanish origin, which may limit generalizability. However, it is promising that similar implementation challenges were documented across different retail contexts [16]. Survey items were also primarily sourced from the nonpublished literature (internal resource), due to a limited number of pragmatic measures available that were considered suitable for a retail context. All items were reviewed for content validity in partnership with practice and retail partners. Also, the survey was distributed during the final weeks of the Pilot Project after an early termination due to customer demand being higher than available funding. This reality led to increased use of the program by customers during this time and may have influenced the challenges reported by MNC retailers. These findings may also provide insights into the potential negative impacts of unsustained public health funding on key partners.

Additionally, MNC retailers’ survey participation was encouraged to take place on the job site and on a work computer, which could have resulted in more favorable responses to survey questions. To mitigate this possibility, survey consent information did include language on confidentiality, including a statement indicating that individuals’ responses would not be shared with coworkers or supervisors. Also, given the quantitative survey design used in this study, the open-ended response options cannot be considered comprehensive; additional explanatory qualitative studies would be needed. Last, the progressive social and political context of Southern California, the strong motivation of MNC to offer public health nutrition programming for customers, and the supportive approach to implementation by UCSD and MNC likely influenced the positive findings in this study. Implementation of similar NIPs within other retail settings or US states may encounter different barriers and enablers; however, the implementation strategies used and identified in this study can serve as a guidepost.

## Conclusion

This study highlights brick-and-mortar retailers’ favorable perceptions of and experiences with the implementation of an NIP using EBT integration. Both the enthusiasm and lessons learned from implementing the Pilot Project are intended to provide a foundation from which to build as NIPs using EBT integration expand to other retail and community contexts. Specifically, the findings in this study can be leveraged by collaborative partners to design and test appropriate implementation strategies intended to support the adoption, implementation, sustainment, and scaling of NIPs using EBT integration in food retail. Insights from this study offer practical guidance for researchers, public health practitioners, food retailers, and policymakers seeking to improve access to healthy foods for SNAP participants nationwide.

## Supplementary Material

ibag020_Supplementary_Data

## Data Availability

Deidentified data from this study are not available in a public archive. Deidentified data from this study may be made available upon reasonable request by emailing the corresponding author.

## Welfare of animals

This article does not contain any studies with animals performed by any of the authors.
