# Supplementary material for: Brick-and-mortar food retail implementation of a nutrition incentive program using Electronic Benefit Transfer integration in Southern California
Source: Transl Behav Med. 2026 May 11;16(1):ibag020. doi: 10.1093/tbm/ibag020 (PMC13748465; doi:10.1093/tbm/ibag020)
Supplement: Supplementary file 1 [file ibag020_supplementary_data.zip › TBM-Supplemental-Table-1.docx]

| Supplemental Table 1. Implementation strategies^a^ used to support the adoption and implementation of a nutrition incentive program using Electronic Benefit Transfer (EBT) integration at the point of sale among 79 Mothers Nutritional Center (MNC) Stores in Southern California, 2024. | |
| --- | --- |
| **Implementation Strategy** | **Description** |
| Choose strategic partner organizations | MNC was selected as an implementing partner for the Pilot Project given their history in supporting local community members through nutrition assistance programs. |
| Design pragmatic programs | The Pilot Project was designed to be fully integrated into Electronic Benefit Transfer (EBT) systems to reduce the burden of nutrition incentive earning and redeeming procedures to improve the reach of the program to all eligible Supplemental Nutrition Assistance Program (SNAP) customers. |
| Engage partners | Advocacy efforts were led by SNAP (CalFresh) participants and key partners, including Nourish California, Fullwell, the San Diego County Childhood Obesity Initiative, and its Community Council. For example, Fulwell scheduled meetings with elected officials at various MNC stores with program partners and participants to educate about the program. Further, Nourish California, with the support of 10,000 program participants, conducted outreach with elected officials. Social media content featuring testimonials were also launched in collaboration with community-based organizations, county health departments, social service agencies, and safety net providers. |
| Leverage funding sources | The Pilot Project was initially funded with $9 million from California’s general fund in 2018, and later supplemented with $9.65 million in 2023 and $10 million in 2024. |
| Meet participants’ needs | The Pilot Project was designed to meet a need to improve food and nutrition security among SNAP (CalFresh) participants by incentivizing fresh fruits and vegetables at the point of sale. |
| Provide resources | UCSD provided MNC retailers with Q+A and FAQ sheets in English and Spanish to support knowledge about the Pilot Project and how it worked. In-store signage and promotional merchandise were also provided to MNC retailers to improve customer awareness about the Pilot Project. Last, language translation tools were provided to help bridge potential communication gaps. |
| Provide training | UCSD delivered trainings to MNC district managers and frontline staff focused on the goals of the Pilot Project and the incentive earning and redemption process. |
| Tailor recruitment strategies | UCSD designed targeted text messages and culturally relevant print and digital materials in English and Spanish used to raise awareness of the Pilot Project among SNAP (CalFresh) participants in California. Public awareness of the Pilot Project was further expanded through television, radio, newspaper, and online media coverage. |

^a^Sourced from the Implementation Strategies Applied in Communities (ISAC) compilation.^29^
