# Supplementary material for: Brick-and-mortar food retail implementation of a nutrition incentive program using Electronic Benefit Transfer integration in Southern California
Source: Transl Behav Med. 2026 May 11;16(1):ibag020. doi: 10.1093/tbm/ibag020 (PMC13748465; doi:10.1093/tbm/ibag020)
Supplement: Supplementary file 1 [file ibag020_supplementary_data.zip › TBM-Supplemental-Table-2.docx]

| Supplemental Table 2. Brick and mortar retailers’ responses to open-ended questions about implementing a nutrition incentive program using Electronic Benefit Transfer (EBT) integration at the point of sale across 79 Mother’s Nutritional Center stores in Southern California, 2024, n=247.^a^ | | | |
| --- | --- | --- | --- |
| **Theme** | **Theme Description** | **Responses n (%)** | **Example Quotation** |
| The Pilot Project contributes to community food and nutrition security. | Retailers viewed the Pilot Project as a valuable community benefit, particularly for enhancing food and nutrition security amid inflation and high living costs, with notable impact among seniors and children. | 127 (51.4%) | *“I am proud to be able to promote this program to the community. My job was to promote it and see the faces when they understood the program and the happiness and relief it brought them. I've been thanked by the people that I've told this program to. I hope it continues in some form. It brought a lot of happiness and fed a lot of people from toddlers to senior citizens*.” |
| Extend the Pilot Project to optimize benefits for customers, communities, and the organization. | Retailers expressed interest in extending the Pilot Project—potentially permanently—to sustain its positive impacts on customers, local communities, and the organization. | 68 (27.5%) | *“Please don’t end the program. It’s amazing how many fruits and vegetables we sold during this time. It shows you how this program helps people eat healthier*.  *Please keep the program alive. It’s a win/win/win for everyone, mainly our customers*.” |
| Enhance incentive tracking capabilities for Pilot Project participants. | Customer challenges in tracking incentive balances—such as lack of awareness or difficulty checking balances—created time constraints for retailers and slowed transactions. Retailers noted that customers often disregarded receipts, requiring cashiers to process transactions to view balances. Although a mobile app was available for checking EBT balances, some retailers suggested developing one, indicating limited awareness of existing tools among both customers and staff. | 54 (21.9%) | *“The entire staff, and the EBT participants would greatly benefit if there could be a way for us to check the balance used. We can only check current balance available in the EBT card, but not how much of the CalFresh Fruit and Vegetable EBT Pilot program was used. We always strive to provide an easy, fast and friendly shopping experience*.” |
| Increase awareness, understanding, and accessibility of the Pilot Project through advertising, promotion, and customer education. | Some retailers reported customer confusion regarding key aspects of the Pilot Project, such as the ability to use the $60 benefit across multiple transactions and how incentives were returned to their EBT cards. Marketing materials occasionally contained unclear language, contributing to misunderstandings and requiring staff to explain the program in-store. Retailers recommended revising promotional wording and establishing a hotline—particularly to support elderly populations. Additionally, four retailers noted that some customers were unaware of the Pilot Project. | 44 (17.8%) | *“I would change the way that the company advertises the project; many of the customers will find only the other customers that post it on social media and no other way. I would recommend that we do a radio and add it to the news for each county the stores are in to indicate what it is about. Next, I would have the company give proper explanations on the ways the program works on the advertisements. Many people come in and expect their EBT to gain money, such as a customer coming in with $10 and spending it, expecting to have a total of $20. Most of the customers that come in expect that to happen, not knowing that it doesn’t work that way*.” |
| Revise incentive transaction rules to enhance checkout efficiency and program impact. | Retailers suggested revising or clarifying incentive transaction rules to better align with store operations. Ten retailers advocated for increasing the incentive cap, while two recommended a decrease. Retailers also proposed removing barriers for customers with low or zero EBT balances, as these individuals often had to make multiple small purchases (e.g., six $10 transactions) to access the full $60 benefit—straining staff capacity, increasing checkout times, and frustrating customers. Additional suggestions included implementing transaction limits (e.g., item caps or designated shopping times) to streamline operations and better serve WIC customers. | 33 (13.4%) | *“If CalFresh is giving away $60 a month to customers who purchase fruits and vegetables, they should be able to purchase fruits and vegetables even if they have no balance on their cards. Most times, customers arrive with only a couple of dollars on their card, making it difficult for them to take their desired produce. Therefore, this also makes it difficult for us because we have to ring up multiple transactions in order to meet the desired amount they would like to take*.” |
| Strengthen staffing capacity, operational support, and infrastructure to more effectively implement the Pilot Project. | A few retailers recommended hiring additional staff to support the growing demands of the Pilot Project, particularly multilingual personnel and shelf stockers. Increased store traffic also highlighted a need for improved layout and infrastructure to accommodate customer volume. Retailers emphasized that clearer communication from upper management could reduce staff confusion during implementation. | 29 (11.7%) | “*We don’t have enough employees to maintain the standards that the company wants. Many team players complain about how it’s too much work for the number of staff; we have customers wait most of the time for 5 mins or more because of that and complain to us or leave the location. Curbside is not supported well. When it comes to both types of consumers, we don’t have enough people to help in store as well as online orders at the same time. There's not enough registers and our store is too small to handle new numbers of people, which makes it very difficult for staff to navigate. This program is great, but we need more space and more people. This program will be better for other larger locations, but for us, it’s very overwhelming.*” |
| Address language barriers between retailers and customers to strengthen implementation and engagement with the Pilot Project. | Some retailers reported challenges communicating with customers who did not speak English or Spanish. To address this, they recommended providing multilingual resources—such as in-store flyers and translation tools—to better convey Pilot Project details and improve customer understanding. | 23 (9.3%) | “*Most of our customers were Vietnamese; language was a big barrier for us. They didn’t have much access to technology or weren’t too advanced in that area, so most of the time we had to be super detailed about how the program works what products applied to the program*.” |
| Expand eligible product categories under the Pilot Project to increase flexibility and customer benefit. | Several retailers recommended expanding eligible items for earning incentives beyond fresh produce. Frozen and canned produce were most frequently suggested, followed by dairy products and eggs. One retailer linked this recommendation to community accessibility concerns, emphasizing the need for broader access to nutritious options. | 14 (5.7%) | “*I understand that this program is to promote healthy eating and encourage more fresh produce in one’s diet, but, a good chunk of our customers are either homeless or do not have a place to prepare their meals. Therefore, possibly adding frozen and canned fruits and veggies to the program can meet the needs of these MNC* *customers*.” |
| Expand training and technical assistance to better equip retailers in implementing the Pilot Project. | A few retailers requested expanded training and technical assistance, including site visits to support store staff. Two employees reported having to learn Pilot Project operations independently. Key areas identified for additional support included translation services and transaction procedures. | 11 (4.5%) | “*I felt we should have had an expert of the program come to each store to explain to us about the program. Even though there was talk about it from managers, we still needed some questions answered. There were things we found out over time on our own about working through the program*.” |

^a^Open-ended survey response options were included to capture additional perspectives related to implementation that may not have been captured via close-ended survey responses. These questions were optional and intentionally broad; thus, these results pertain to those who chose to respond to an open-ended question and reflect the varied topics brought forward by retailers.
