# Supplementary material for: Brick-and-mortar food retail implementation of a nutrition incentive program using Electronic Benefit Transfer integration in Southern California
Source: Transl Behav Med. 2026 May 11;16(1):ibag020. doi: 10.1093/tbm/ibag020 (PMC13748465; doi:10.1093/tbm/ibag020)
Supplement: Supplementary file 1 [file ibag020_supplementary_data.zip › TBM-Supplemental-File-1.docx]

**Supplemental File 1**: Survey used to evaluate Mother’s Nutritional Center (MNC) leadership and staff implementation of the CalFresh Fruit and Vegetable EBT Pilot Project across 79 stores in Southern California, 2024.

*Module 1: Social and Demographic Characteristics*

**Module explanation:** Information in Module 1 helps us understand more about the retailers carrying out the program and how perspectives may vary by location, position details, and /or social and demographic characteristics.

*First, we would like to learn about you and your position at MNC.*

1. What is your age? [All respondents]

- ___________ (open-ended, whole numbers only)
- Prefer not to answer

2. How do you describe yourself? [All respondents]

- Female
- Male
- Non-binary/third gender
- Prefer not to answer

3. How would you describe your racial or ethnic background? Check all that apply. [All respondents]

- American Indian or Alaska Native
- Asian
- Black or African American
- Hispanic, Latino/a, or Spanish origin
- Other Pacific Islander
- White
- Some other race: _________________
- Prefer not to answer

4. For how long have you worked at MNC? [All respondents]

- Less than one month
- Less than one year, but more than one month
- Between 1 and 2 years
- Between 2 and 3 years
- 3 years or more
- 5 years or more
- 10 years or more
- Prefer not to answer

5. What is your current job title? [All respondents]

- Owner
- Director (Operations Director, Marketing Director, Buying Director, IT Director, Special Projects Director)
- District Manager
- Marketing/Outreach Manager
- Manager
- Assistant Manager
- 3rd Person
- Crew Leader
- Cashier
- Produce Attendant
- Stocker

6. In what zip code is your MNC store located? [Managers; Other Staff]

- ___________ (open-ended, whole numbers only)
- Prefer not to answer
- I don’t know and/or not applicable

--------------------------------

*Module 2: EPIS Leadership*

**Module explanation:** Information in Module 2 helps us to understand leadership support, both at a higher level and at a store level, along four characteristics found to be beneficial for implementing programs in organizations: proactive; knowledgeable; supportive; and perseverant.

*Next, we would like to learn about your support for the CalFresh Fruit and Vegetable EBT Pilot Project as MNC leadership/management.*

*Please indicate your level of agreement with each statement.*

[Higher Level Leadership; Managers]

7. I have provided staff with the tools and resources to help carry out the CalFresh Fruit and Vegetable EBT Pilot Project in MNC stores/my store.

- Completely disagree
- Disagree
- Neither agree nor disagree
- Agree
- Completely agree
- Prefer not to answer
- I don’t know and/or not applicable

8. I have removed obstacles to carrying out the CalFresh Fruit and Vegetable EBT Pilot Project in MNC stores/my store.

- Completely disagree
- Disagree
- Neither agree nor disagree
- Agree
- Completely agree
- Prefer not to answer
- I don’t know and/or not applicable

9. I have established clear standards for carrying out the CalFresh Fruit and Vegetable EBT in MNC stores/my store.

- Completely disagree
- Disagree
- Neither agree nor disagree
- Agree
- Completely agree
- Prefer not to answer
- I don’t know and/or not applicable

10. I am knowledgeable about the CalFresh Fruit and Vegetable EBT Pilot Project.

- Completely disagree
- Disagree
- Neither agree nor disagree
- Agree
- Completely agree
- Prefer not to answer
- I don’t know and/or not applicable

11. I can answer staff’s questions about the CalFresh Fruit and Vegetable EBT Pilot Project.

- Completely disagree
- Disagree
- Neither agree nor disagree
- Agree
- Completely agree
- Prefer not to answer
- I don’t know and/or not applicable

12. I know what I am talking about when it comes to the CalFresh Fruit and Vegetable EBT Pilot Project.

- Completely disagree
- Disagree
- Neither agree nor disagree
- Agree
- Completely agree
- Prefer not to answer
- I don’t know and/or not applicable

13. I recognize and appreciate employee efforts toward successfully carrying out the CalFresh Fruit and Vegetable EBT Pilot Project.

- Completely disagree
- Disagree
- Neither agree nor disagree
- Agree
- Completely agree
- Prefer not to answer
- I don’t know and/or not applicable

14. I support employee efforts to learn more about the CalFresh Fruit and Vegetable EBT Pilot Project.

- Completely disagree
- Disagree
- Neither agree nor disagree
- Agree
- Completely agree
- Prefer not to answer
- I don’t know and/or not applicable

15. I support employee efforts to carry out the CalFresh Fruit and Vegetable EBT Pilot Project.

- Completely disagree
- Disagree
- Neither agree nor disagree
- Agree
- Completely agree
- Prefer not to answer
- I don’t know and/or not applicable

16. I persist through the ups and downs of carrying out the CalFresh Fruit and Vegetable EBT Pilot Project.

- Completely disagree
- Disagree
- Neither agree nor disagree
- Agree
- Completely agree
- Prefer not to answer
- I don’t know and/or not applicable

17. I continue through the challenges of carrying out the CalFresh Fruit and Vegetable EBT Pilot Project.

- Completely disagree
- Disagree
- Neither agree nor disagree
- Agree
- Completely agree
- Prefer not to answer
- I don’t know and/or not applicable

18. I respond to critical issues about carrying out the CalFresh Fruit and Vegetable EBT Pilot Project by openly and effectively addressing the problem(s).

- Completely disagree
- Disagree
- Neither agree nor disagree
- Agree
- Completely agree
- Prefer not to answer
- I don’t know and/or not applicable

[Other Staff]

*Next, we would like to learn about MNC* *store leadership/management support for the CalFresh Fruit and Vegetable EBT Pilot Project in your store from your perspective.*

*Please indicate your level of agreement with each statement.*

19. MNC leadership/management has provided the tools and resources to help carry out the CalFresh Fruit and Vegetable EBT Pilot Project.

- Completely disagree
- Disagree
- Neither agree nor disagree
- Agree
- Completely agree
- Prefer not to answer
- I don’t know and/or not applicable

20. MNC leadership/management has removed obstacles to carrying out the CalFresh Fruit and Vegetable EBT Pilot Project.

- Completely disagree
- Disagree
- Neither agree nor disagree
- Agree
- Completely agree
- Prefer not to answer
- I don’t know and/or not applicable

21. MNC leadership/management has established clear store standards for carrying out the CalFresh Fruit and Vegetable EBT Pilot Project.

- Completely disagree
- Disagree
- Neither agree nor disagree
- Agree
- Completely agree
- Prefer not to answer
- I don’t know and/or not applicable

22. MNC leadership/management is knowledgeable about the CalFresh Fruit and Vegetable EBT Pilot Project.

- Completely disagree
- Disagree
- Neither agree nor disagree
- Agree
- Completely agree
- Prefer not to answer
- I don’t know and/or not applicable

23. MNC leadership/management can answer my questions about the CalFresh Fruit and Vegetable EBT Pilot Project.

- Completely disagree
- Disagree
- Neither agree nor disagree
- Agree
- Completely agree
- Prefer not to answer
- I don’t know and/or not applicable

24. MNC leadership/management knows what they are talking about when it comes to the CalFresh Fruit and Vegetable EBT Pilot Project.

- Completely disagree
- Disagree
- Neither agree nor disagree
- Agree
- Completely agree
- Prefer not to answer
- I don’t know and/or not applicable

25. MNC leadership/management recognizes and appreciates employee efforts toward successfully carrying out the CalFresh Fruit and Vegetable EBT Pilot Project.

- Completely disagree
- Disagree
- Neither agree nor disagree
- Agree
- Completely agree
- Prefer not to answer
- I don’t know and/or not applicable

26. MNC leadership/management supports employee efforts to learn more about the CalFresh Fruit and Vegetable EBT Pilot Project.

- Completely disagree
- Disagree
- Neither agree nor disagree
- Agree
- Completely agree
- Prefer not to answer
- I don’t know and/or not applicable

27. MNC leadership/management supports employee efforts to carry out the CalFresh Fruit and Vegetable EBT Pilot Project.

- Completely disagree
- Disagree
- Neither agree nor disagree
- Agree
- Completely agree
- Prefer not to answer
- I don’t know and/or not applicable

28. MNC leadership/management persists through the ups and downs of carrying out the CalFresh Fruit and Vegetable EBT Pilot Project.

- Completely disagree
- Disagree
- Neither agree nor disagree
- Agree
- Completely agree
- Prefer not to answer
- I don’t know and/or not applicable

29. MNC leadership/management continues through the challenges of carrying out the CalFresh Fruit and Vegetable EBT Pilot Project.

- Completely disagree
- Disagree
- Neither agree nor disagree
- Agree
- Completely agree
- Prefer not to answer
- I don’t know and/or not applicable

30. MNC leadership/management responds to critical issues about carrying out the CalFresh Fruit and Vegetable EBT Pilot Project by openly and effectively addressing the problem(s).

- Completely disagree
- Disagree
- Neither agree nor disagree
- Agree
- Completely agree
- Prefer not to answer
- I don’t know and/or not applicable

--------------------------------

*Module 3: EPIS Organizational Characteristics*

**Module explanation:** Information in Module 3 helps us to understand how well program implementation aligns with store business models and procedures, as this can influence program success initially and over time.

*Next, we would like to learn about the alignment of the* *CalFresh Fruit and Vegetable EBT Pilot Project with MNC’s mission, values, and store processes/procedures.*

31. Why did MNC choose to participate in the CalFresh Fruit and Vegetable EBT Pilot Project? *Check all that apply.* [Higher Level Leadership]

- To benefit customers
- To increase sales of fruits and vegetables
- To increase sales of other items
- To be a part of something new
- We were asked to join
- We know other stores who joined
- Another reason
  - Please describe: ____________
- Prefer not to answer
- I don’t know and/or not applicable

32. The **cost** investment for MNC to carry out the CalFresh Fruit and Vegetable EBT Pilot Project was worthwhile. [Higher Level Leadership]

- Completely disagree
- Disagree
- Neither agree nor disagree
- Agree
- Completely Agree
- Prefer not to answer
- I don’t know and/or not applicable

33. The **time** investment for MNC to carry out the CalFresh Fruit and Vegetable EBT Pilot Project was worthwhile. [Higher Level Leadership]

- Completely disagree
- Disagree
- Neither agree nor disagree
- Agree
- Completely Agree
- Prefer not to answer
- I don’t know and/or not applicable

34. MNC has a good relationship with the community that makes it easier to carry out the CalFresh Fruit and Vegetable EBT Pilot Project. [All respondents]

- Completely disagree
- Disagree
- Neither agree nor disagree
- Agree
- Completely Agree
- Prefer not to answer
- I don’t know and/or not applicable

35. Is there anything you would change about the CalFresh Fruit and Vegetable EBT Pilot Project to better align with **MNC’s mission or values**? [All respondents]

- Yes
  - Please describe: ____________
- No
- Prefer not to answer
- I don’t know and/or not applicable

36. Is there anything you would change about the CalFresh Fruit and Vegetable EBT Pilot Project to better align with **MNC’s store processes/procedures**? [All respondents]

- Yes
  - Please describe: ____________
- No
- Prefer not to answer
- I don’t know and/or not applicable

37. Is there anything you would change about the CalFresh Fruit and Vegetable EBT Pilot Project to **better meet the needs of MNC’s customers**? [All respondents]

- Yes
  - Please describe: ____________
- No
- Prefer not to answer
- I don’t know and/or not applicable

--------------------------------

*Module 4: EPIS Quality and Fidelity Monitoring and Support*

**Module explanation:** Information in Module 4 helps us to understand what is helping (or what may be needed to help) implement programs in stores, as providing ongoing technical assistance or support may help to support program delivery.

*Next, we would like to learn about available or needed supports to help to carry out the* CalFresh Fruit and Vegetable EBT *Pilot Project in your store.*

38. How would you rate your overall experience with marketing and promotion of the CalFresh Fruit and Vegetable EBT Pilot Project in your store? [Managers, Other Staff]

- Very negative
- Negative
- Neutral
- Positive
- Very positive
- Prefer not to answer
- I don’t know and/or not applicable

39. The provided trainings, resources, and materials helped to prepare me/my team to carry out the CalFresh Fruit and Vegetable EBT Pilot Project. [Managers, Other Staff]

- Completely disagree
- Disagree
- Neither agree nor disagree
- Agree
- Completely Agree
- Prefer not to answer
- I don’t know and/or not applicable

40. Do you or your team need additional support to better carry out the CalFresh Fruit and Vegetable EBT Pilot Project (e.g., communication, resources, technical assistance, training, site visits)? [Managers, Other Staff]

- Yes
  - Please describe: ____________
- No
- Prefer not to answer
- I don’t know and/or not applicable

--------------------------------

*Module 5: EPIS Organizational Staffing Processes*

**Module explanation:** Module 5 helps us to understand how implementing the program impacts employee time and position requirements, as this can influence the success of implementation initially and over time.

*Next, we would like to learn about how carrying out the CalFresh Fruit and Vegetable EBT Pilot Project impacts store staff.*

41. MNC has enough staff with the right skills to carry out the CalFresh Fruit and Vegetable EBT Pilot Project. [Managers, Other Staff]

- Completely disagree
- Disagree
- Neither agree nor disagree
- Agree
- Completely Agree
- Prefer not to answer
- I don’t know and/or not applicable

42. How easy or difficult is it to explain how the CalFresh Fruit and Vegetable EBT Pilot Project works to customers? [Managers, Other Staff]

- Very difficult
- Difficult
- Neutral
- Easy
- Very easy
- Prefer not to answer
- I don’t know and/or not applicable

43. How easy or difficult is it to explain the types of fruits and vegetables that qualify for the CalFresh Fruit and Vegetable EBT Pilot Project? [Managers, Other Staff]

- Very difficult
- Difficult
- Neutral
- Easy
- Very easy
- Prefer not to answer
- I don’t know and/or not applicable

44. How easy or difficult is it to explain the differences between the CalFresh Fruit and Vegetable EBT Pilot Project and other federal/state store programs (e.g., WIC, CalFresh, cash EBT)? [Managers, Other Staff]

- Very difficult
- Difficult
- Neutral
- Easy
- Very easy
- Prefer not to answer
- I don’t know and/or not applicable

45. What kinds of questions have customers asked about the CalFresh Fruit and Vegetable EBT Pilot Project? *Check all that apply.* [All respondents]

- Questions about how the program works in the store (e.g., how to participate in the CalFresh Fruit and Vegetable EBT Pilot Project at MNC versus farmers markets, how to use incentives).
- Questions about program logistics (e.g., eligible purchase items)
- Questions about the pilot project ending
- Questions about incentive/EBT balance (e.g., balance amounts on receipt or ebtEDGE phone app, incentive delivery)
- Questions about signing up for CalFresh EBT or similar programs (e.g., WIC)
- Questions about filling out the CDSS online program participant survey
- Other, please specify: _________
- Customers did not ask me any questions about the program
- Prefer not to answer
- I don’t know and/or not applicable

46. How easy or difficult is it to ring up or handle transactions for the CalFresh Fruit and Vegetable EBT Pilot Project? [Managers, Other Staff]

- Very difficult
- Difficult
- Neutral
- Easy
- Very easy
- Prefer not to answer
- I don’t know and/or not applicable

47. Compared to transactions for repeat CalFresh Fruit and Vegetable EBT Pilot Program customers, how much extra time would you say that it takes, on average, to process a transaction **for a customer who is new to the program**? [Managers, Other Staff]

- No more time
- Less than 30 seconds
- Between 30 seconds to 2 minutes
- Between 2 minutes to 5 minutes
- More than 5 minutes
- Prefer not to answer
- I don’t know and/or not applicable

--------------------------------

*Module 6: EPIS Individual Characteristics*

**Module explanation:** Module 6 helps us to understand individual attitudes, beliefs, and perceptions about the program, given this can influence buy-in for supporting program implementation initially and over time.

*Lastly, we would like to learn about how individual beliefs and perceptions can impact carrying out the* *CalFresh Fruit and Vegetable EBT Pilot Project.*

48. Overall, how would you rate your experience carrying out the CalFresh Fruit and Vegetable EBT Pilot Project? [Managers, Other Staff]

- Very negative
- Negative
- Neutral
- Positive
- Very positive
- Prefer not to answer
- I don’t know and/or not applicable

49. On a scale of 1 to 10, how likely is it that you would recommend the CalFresh Fruit and Vegetable EBT Pilot Project to a friend or colleague? [All respondents]

- 1 (Not at all likely)
- 2
- 3
- 4
- 5
- 6
- 7
- 8
- 9
- 10 (Extremely likely)
- Prefer not to answer
- I don’t know and/or not applicable

50. It is important to support programs like the CalFresh Fruit and Vegetable EBT Pilot Project that promote access to fruits and vegetables. [All respondents]

- Completely disagree
- Disagree
- Neither agree nor disagree
- Agree
- Completely Agree
- Prefer not to answer
- I don’t know and/or not applicable

51. Participation in the CalFresh Fruit and Vegetable EBT Pilot Project gives me an opportunity to give back to my community. [All respondents]

- Completely disagree
- Disagree
- Neither agree nor disagree
- Agree
- Completely Agree
- Prefer not to answer
- I don’t know and/or not applicable

52. I would support CalFresh Fruit and Vegetable EBT participation in a program similar to the CalFresh Fruit and Vegetable EBT Pilot Project again. [All respondents]

- Completely disagree
- Disagree
- Neither agree nor disagree
- Agree
- Completely Agree
- Prefer not to answer
- I don’t know and/or not applicable

--------------------------------

*Module 7: Closing Question*
**Module explanation:** An open-ended question at the end of a survey provides an opportunity for someone to share beyond what we have asked about on the survey. It also provides a space, if needed, to expand on a concept that was asked in a selection response option.

53. Would you like to share anything else with us (e.g., what you like, what you dislike) about the CalFresh Fruit and Vegetable EBT Pilot Project? [All respondents]

- Yes
  - Please describe: ____________
- No
- Prefer not to answer
- I don’t know and/or not applicable

*Thank you for your responses to our questions. Your insight is valuable.*
